# Choledochoplasty with Gallbladder Wall Free Flap: A Novel Technique for Large Bile Duct Defects from Mirizzi Syndrome in High-Risk Patients—A Case Report and Literature Review

**DOI:** 10.1155/2019/4615484

**Published:** 2019-07-30

**Authors:** Louis F. Chai, Gary S. Xiao

**Affiliations:** Division of Multi-Organ Transplantation and Hepato-Pancreatic-Biliary Surgery, Department of Surgery, Drexel University College of Medicine, Hahnemann University Hospital, Philadelphia PA 19102, USA

## Abstract

**Background:**

Cholecystectomies are almost universally performed laparoscopically with complication rates similar to open surgery. Possible complications include bleeding and damage to surrounding structures. These often require intervention to repair the damage immediately when recognized intraoperatively or postoperatively. These injuries can cause significant morbidity and mortality, and additional interventions further compound this, especially for high-risk patients. All attempts should be made to a lower risk while performing the safest operation and addressing complications appropriately. We present a case of a surgically high-risk patient who underwent an attempted laparoscopic, converted to open, cholecystectomy for Mirizzi syndrome, during which a biliary defect was found and repaired with a novel technique of choledochoplasty with a gallbladder wall free flap.

**Case:**

An 82-year-old female with abdominal pain was diagnosed with a cholecystocholedochal fistula from chronic cholecystitis and Mirizzi syndrome. During cholecystectomy, a large common bile duct defect was noted, and given intraoperative instability, the repair was completed using a gallbladder wall free flap. Postoperatively, the patient recovered well through a 4.5-year follow-up.

**Conclusion:**

Complications from laparoscopic cholecystectomy are rare but may result in additional interventions. For patients who are high-risk surgical candidates, gallbladder wall free flap choledochoplasty should be considered to avoid additional morbidity and mortality.

## 1. Introduction

Mirizzi syndrome is a rare complication of chronic cholecystitis that can lead to obstructive jaundice from compression or fibrosis of the adjacent common hepatic or common bile ducts. Severe cases can lead to the formation of fistulas between the gallbladder, cystic duct, common hepatic ducts, and common bile ducts. The implication of this rare phenomenon is greatest in the operative management of cholecystitis as the critical view of safety is difficult, if not impossible, to obtain, and there are high incidences of biliary tree injuries. Though simple cholecystectomies may be completed in mild cases, partial cholecystectomies may be required in order to prevent such injuries, though they are not always avoidable. With significant defects, repair with biliary-enteric anastomoses such as a Roux-en-Y hepaticojejunostomy or choledochoplasty is considered. In the world literature, a combination of partial cholecystectomy and repair of the damaged common channels with a pedicled gallbladder flap has been described. We present here a case report of a choledochoplasty with a novel, never before reported gallbladder wall free flap in the case of an unstable patient with multiple medical comorbidities that would make a hepaticojejunostomy a high-risk operation. We believe that the adaptation of this technique may be beneficial in reducing the operative complications on patients with Mirizzi syndrome.

## 2. Case

The patient was an 82-year-old female who is a nursing home resident presented to an outside hospital with a two-week history of jaundice that had progressively worsened. She was brought to the emergency department and found to have hyperbilirubinemia with a total bilirubin of 10.7 and a direct bilirubin of 8.7. She was afebrile, and white blood cell count was 8.9. A CT scan of her abdomen and a MRCP were diagnostic for Mirizzi syndrome, revealing large stones compressing the common bile duct (CBD) with proximal dilation. She was transferred to our hospital for a higher level of care with hepato-pancreatico-biliary surgeons. Of note, her past medical history was significant for morbid obesity with a BMI of 42, dementia, diabetes mellitus, hypothyroidism, deep vein thrombosis (DVT), depression, anemia, tobacco use, and congestive heart failure (CHF). Her surgical history consisted of an appendectomy.

**Figure 1:**
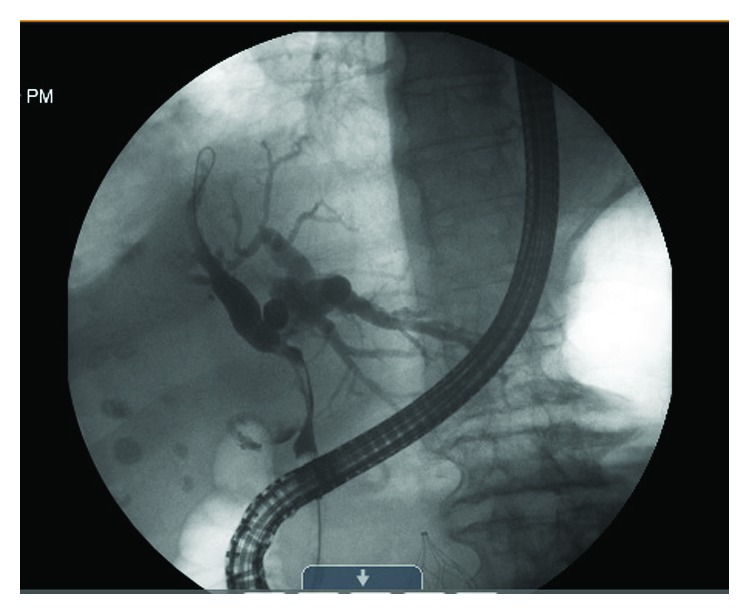
Preoperative ERCP demonstrating a significantly dilated intrahepatic biliary tree, external compression of the extrahepatic biliary ducts, and large filling defect of the cystic duct and gall bladder.

Upon arrival at our hospital, the patient underwent an endoscopic retrograde cholangiopancreatography (ERCP) with the removal of small stones in the common bile duct, sphincterotomy, and two stent placements with ends terminating in the distal right and left hepatic ducts. However, despite this, there was still tapering in the common hepatic and common bile ducts noted from extrinsic compression from the large stones in the gallbladder and a significant filling defect through the cystic duct (Figure 1). Additionally, the patient developed a fever and leukocytosis and persistently elevated bilirubin, with the most likely cause being an obstruction of the biliary stents causing persistent cholangitis and cholecystitis. The ERCP was repeated, and a third stent was placed. The patient's fever was controlled, and the bilirubin began to trend down.

**Figure 2:**
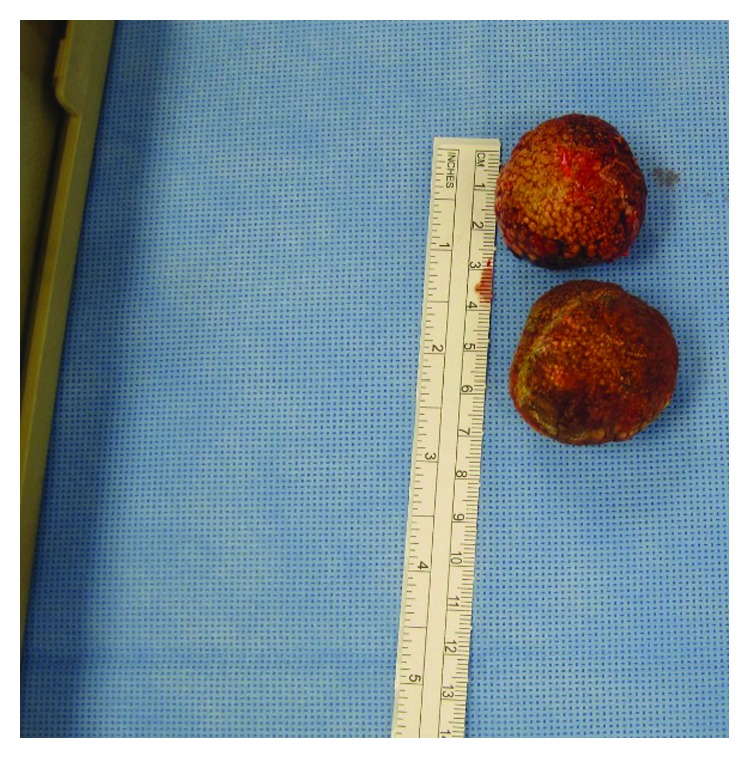
Two large gall stones were dissected free from the gallbladder and surrounding structures after a partial cholecystectomy was performed. Each stone measured approximately 3-4 centimeters in diameter.

Once the patient was stabilized, she was taken to the operating room for a cholecystectomy. The procedure began laparoscopically, but dense adhesions in the right upper quadrant involving the liver, gall bladder, omentum, and transverse colon prevented the development of adequate and safe dissection planes, and thus, the procedure was converted to open. The lysis of adhesions continued to be difficult, but once the gallbladder was dissected out, two large, firm masses were palpated within the fundus and the neck of the gallbladder. Given the difficulty with the anatomy to safely do a cholecystectomy with the gallstones en bloc, the decision was made at this time to do a partial cholecystectomy with the removal of the stones. The anterior gallbladder wall was first opened near the fundus, and the gallbladder wall was first dissected off the stone and then the stone from the posterior wall. When removed, the stone measured approximately 3.5 centimeters (Figure 2), and pus and necrotic tissue were found within the gallbladder.

Attention was then turned to the neck of the gallbladder where the anterior wall of the gallbladder was again incised, revealing another a large 4-centimeter stone (Figure 2) that was dissected off the posterior wall and removed. It was noted at this time that there was significant arterial bleeding with a loss of approximately 300 cc of blood and the development of mild hypotension and tachycardia. Given the new symptoms and the patient's baseline hemoglobin of 8.4, the patient received two units of red blood cells intraoperatively. The arterial bleeding was controlled with sutures, and attention was turned to the biliary tree upon which a defect in the common hepatic duct and the common bile duct was noted, presumably where the cystic duct previously entered and from the cholecystocholedochal fistula formation. The defect measured approximately three centimeters in length and eight millimeters in width after debridement of necrotic tissue.

**Figure 3:**
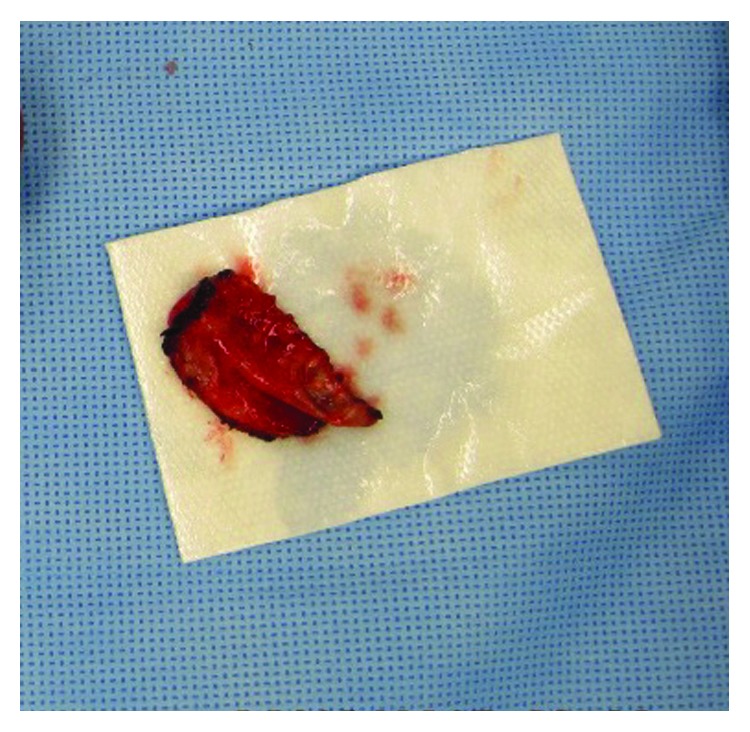
Free flap of the gallbladder wall after partial cholecystectomy was performed. The luminal surface is demonstrated here with total flap size measuring approximately 4 centimeters in length and 2.5 centimeters in width.

Given the fragility of the tissue and the size of CBD defect, it was not closed primarily. However, the patient's hemodynamic instability and overall health status made a biliary-enteric anastomosis a very high-risk operation with significant morbidity and mortality for the patient. Thus, the decision was made to use a dissected, viable gallbladder wall piece to form a free flap to repair the defect in the biliary tree (Figure 3). The mucosal surface of the gall bladder wall was juxtaposed to the lumen of the common ducts and secured in place with a running 5-0 PDS suture. The remnant gallbladder stump was closed and oversewn in a continuous fashion. The area was extensively irrigated given the pus and necrosis that was discovered during the cholecystectomy. One drain was placed within the closed gallbladder remnant and the second one outside the closed gallbladder remnant. The abdomen was then closed in the usual manner.

**Figure 4:**
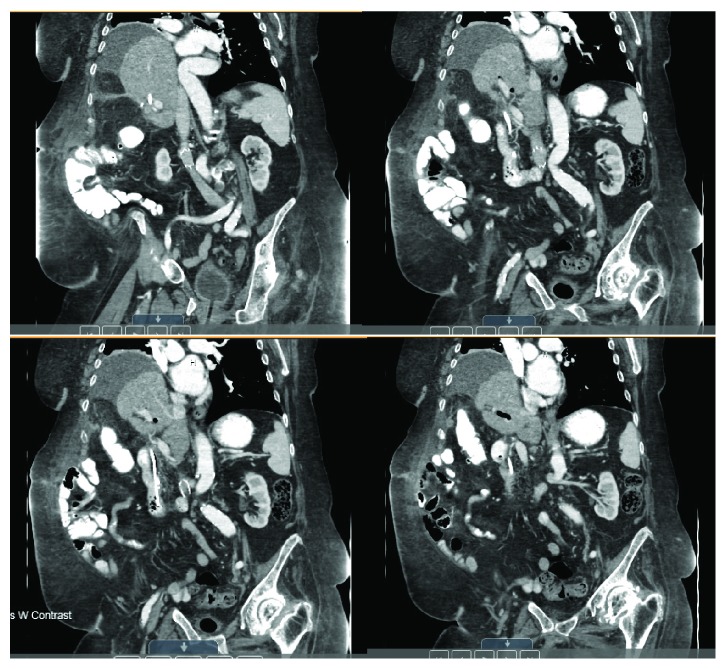
Postoperative follow-up imaging revealing no intrahepatic biliary dilation and a patent extrahepatic biliary system and stent (top left, clockwise, anterior to posterior).

Immediately, postoperatively, the patient was managed in the intensive care unit given her instability during the operation but was transferred to a step down unit shortly after on postoperative day two. She was observed off of antibiotics with normalization of leukocytosis, remained afebrile and hemodynamically stable, and had continuously down trending bilirubin levels throughout the remainder of her hospital stay. Clinically, she recovered well and was stable for discharge on postoperative day seven with drains remaining in place and to be removed on a follow-up as an outpatient. On a subsequent follow-up, the patient was noted to remain clinically stable and follow-up CT scans revealed no intrahepatic biliary duct dilation, a normal caliber common bile duct, no intraluminal filling defect, a patent stent, and no biliary leak (Figure 4). Laboratory evaluation revealed no leukocytosis and normalization of liver enzymes and total bilirubin levels. Given these results, her surgical drains were removed, but her biliary stents remained in place, and despite multiple contact points and counseling to have a follow-up with the gastroenterologists for ERCP and removal, she was noncompliant and the stents remain in place to date. She however remains stable and healthy during her one follow-up appointment as of this writing and continues to be monitored closely with phone check-ins with no evidence of further hepatobiliary symptoms or complaints of incisional hernias.

## 3. Discussion

Complications resulting from cholecystectomies may result from aberrant anatomy, technical error, or inflammatory conditions such as Mirizzi syndrome [1]. Surgical options may be to perform an open procedure instead for easier dissection and intraoperative cholangiogram to better delineate anatomy [2–4]. In certain situations, such as with cholecystocholedochal fistulas in our patient, injuries to adjacent structures may be unavoidable to complete the procedure. For such scenarios, therapeutic options must be considered carefully, taking into account the nature of the complication and the patient's clinical status. Therapeutic options are then considered depending on the injury type, classically based on the Strasberg and Bismuth classifications [5–9].

Our patient's injury was the most consistent with a Strasberg Type E and Bismuth Type 1 classification given the 3-centimeter defect, presumably where the cystic duct previously entered the common channels and greater than 2 centimeters from the confluence of the right and left hepatic ducts. Surgical reconstruction in this scenario would be an end-to-side Roux-en-Y hepaticojejunostomy with drain placement. However, given the significant comorbidities and hemodynamic instability of the patient during the surgery, this likely would have resulted in significant postoperative complications. Given this, other therapeutic options were considered and choledochoplasty was performed using a free gallbladder wall flap to restore continuity to the biliary channel as a much-less invasive procedure.

Choledochoplasty is a surgical option that has been described in the world literature as a surgical option for common bile duct damage [10–15]. These are generally performed with a partial cholecystectomy and choledochoplasty using a gallbladder flap, but the technique that is described in these reports uses a pedicle flap whereas in our case, we performed the first known and described case using a free flap from the removed gallbladder wall to repair the common channel defect. The mucosal side of the gallbladder was used as the luminal surface, and the flap was sewn directly over the defect as a patch with drains left in the area of repair. The patient remains stable and asymptomatic 4.5 years after reconstruction.

Much like a Graham patch for a perforated duodenal ulcer, the gallbladder free flap likely forms an area of fibrosis and scarring in the area of healing, thus establishing a continuous conduit once fully healed. The free flap covers the defect and restores the continuity in the common channel, allowing passage of bile to the duodenum if a pedicle flap is not available or the bile duct defect was found after the gallbladder was removed. Additionally, this avoids the technical difficulty of mobilizing a pedicle that is both well perfused and tension free. Likewise, it is an operative technique that should be considered in a patient who may not be able to tolerate mobilization of large segments of bowel or is at a high risk for failure of biliary-enteric anastomoses. In considering the patient's perioperative morbidity and mortality, such an operative technique may help to reduce the risk of suffering harm during a complicated cholecystectomy while at the same time provide a surgical option with a risk that is acceptable for such patients. Thus, in the settings of significant medical comorbidities and intraoperative instability, a pedicle gallbladder flap not being available, or the gallbladder was already removed, a gallbladder free flap should be considered for the repair of a damaged common bile duct or hepatic duct during cholecystectomy. Our case report highlights that this can be a successful operation that avoids the complications associated with other reconstructive techniques.
